# Quantification of heat shock proteins in the posterior interosseous nerve among subjects with type 1 and type 2 diabetes compared to healthy controls

**DOI:** 10.3389/fnins.2023.1227557

**Published:** 2023-08-08

**Authors:** Erik Ising, Emma Åhrman, Niels O. B. Thomsen, Anna Åkesson, Johan Malmström, Lars B. Dahlin

**Affiliations:** ^1^Department of Clinical Sciences—Pediatric Endocrinology, Lund University, Malmö, Sweden; ^2^Department of Emergency and Internal Medicine, Skåne University Hospital, Malmö, Sweden; ^3^Division of Infection Medicine, Department of Clinical Sciences, Faculty of Medicine, Lund University, Lund, Sweden; ^4^Department of Translational Medicine—Hand Surgery, Lund University, Malmö, Sweden; ^5^Department of Hand Surgery, Skåne University Hospital, Malmö, Sweden; ^6^Clinical Studies Sweden—Forum South, Skåne University Hospital, Lund, Sweden; ^7^Department of Biomedical and Clinical Medicine, Linköping University, Linköping, Sweden

**Keywords:** heat shock proteins, diabetes, type 1 diabetes, type 2 diabetes, neuropathy, proteomics

## Abstract

**Introduction:**

Diabetic peripheral neuropathy (DPN) is a common complication of both type 1 (T1D) and type 2 diabetes (T2D). No cure for DPN is available, but several potential targets have been proposed for treatment. Heat shock proteins (HSPs) are known to respond to both hyper- and hypoglycemia. DPN can be diagnosed using electrophysiology and studied using peripheral nerve biopsies.

**Aim:**

This study aimed to analyze the presence and patterns of HSPs in peripheral nerve biopsies from subjects with T1D, T2D, and healthy controls.

**Methods:**

Posterior interosseous nerves (PIN) from a total of 56 subjects with T1D (*n* = 9), with T2D (*n* = 24), and without diabetes (i.e., healthy controls, *n* = 23) were harvested under local anesthesia and prepared for quantitative mass spectrometry analysis. Protein intensities were associated with electrophysiology data of the ulnar nerve and morphometry of the same PIN, and differences in protein intensities between groups were analyzed.

**Results:**

In total, 32 different HSPs were identified and quantified in the nerve specimens. No statistically significant differences were observed regarding protein intensities between groups. Furthermore, protein intensities did not correlate with amplitude or conduction velocity in the ulnar nerve or with the myelinated nerve fiber density of PIN.

**Conclusion:**

Quantitative proteomics can be used to study HSPs in nerve biopsies, but no clear differences in protein quantities were observed between groups in this cohort.

## 1. Introduction

Diabetes is a global pandemic, with an estimated prevalence of approximately 537 million affected people, diagnosed either as type 1 diabetes (T1D) or type 2 diabetes (T2D) (IDF, 2021). Numbers are expected to rise over the years to come. With an increase in prevalence, also the socioeconomic burden on healthcare systems, connected to the management of diabetes and its complications, will escalate.

Neuropathies and nerve compression disorders, such as carpal tunnel syndrome and ulnar nerve compression at the elbow, are common complications to both T1D and T2D (Rydberg et al., 2020), with distal symmetric polyneuropathy, i.e., diabetic peripheral neuropathy (DPN), being the most common complication (Feldman et al., 2019). DPN is closely connected to the development of diabetic foot ulcers, is known to decrease the quality of life in the affected people, and causes great monetary loss (Feldman et al., 2019; Kerr et al., 2019). Diabetes with its neuropathy may also cause peripheral nerves to be more susceptible to nerve compression disorders (Upton and McComas, 1973). The gold-standard method of diagnosing DPN is based on an abnormal nerve conduction study, i.e., electrophysiology (Tesfaye et al., 2010). Although much is known about DPN and its pathogenesis, a lot remains to be explained. Hyperglycemia, dyslipidemia, and insulin resistance have been shown to initiate co-existing pathways, such as the protein kinase C (PKC), polyol, advanced glycation end products (AGE), and hexosamine pathways, which together contribute to inflammation, increased oxidative stress, mitochondrial dysfunction, and altered gene expressions (Feldman et al., 2017, 2019). However, since enhanced glucose control has been shown to be effective in preventing DPN among T1D subjects but has limited effect on DPN in T2D, the differences of DPN in T1D compared to T2D have been emphasized (Callaghan et al., 2012; Jaiswal et al., 2017).

Emerging evidence suggests an essential role for HSPs in neural physiology in the central nervous system (Stetler et al., 2010; de Los Reyes and Casas-Tintó, 2022). In addition to the pathogenic pathways mentioned above, the impact of heat shock proteins (HSPs) has been described in the development of DPN (Feldman et al., 2019). Targeting HSPs has been proposed as a promising strategy in the treatment of DPN in mice by reducing oxidative stress and improving mitochondrial function (Farmer et al., 2012; Urban et al., 2012; de Los Reyes and Casas-Tintó, 2022). HSPs are molecular chaperones mainly serving to maintain cellular homeostasis by folding new, and old misfolded, proteins into functional proteins and clearing misfolded proteins by initiating their degradation (Chaudhury et al., 2021). Following cellular stress, the transcription of HSPs may be upregulated by the transcription factor heat shock factor-1 to reduce the damaging impact of cellular stress (Chaudhury et al., 2021).

Biopsy of the sural nerve represents the gold standard for the study of the morphometry of nerve fibers in DPN (Malik, 2014). Previous studies have been able to show early signs of DPN in the sural nerve among subjects with T1D or T2D (Malik et al., 2005). The changes included paranodal and segmental de- and re-myelination, but initially without axonal degeneration, indicating Schwann cells to be a key target in DPN (Malik et al., 2005). Furthermore, de- and re-generation of small nerve fibers were observed (Malik et al., 2005). Studies on biopsies of the posterior interosseous nerve (PIN), located uncompressed at the dorsal aspect of the forearm just proximal to the wrist, have been demonstrated as an alternative to a sural nerve biopsy in DPN due to its accessibility and minimal postoperative complications (Thomsen et al., 2009a,b). However, PIN biopsies may potentially show less structural pathology compared to the sural nerve in the same subject since PIN is potentially in a more vascularized limb and not as distally located (Dahlin; personal observation). DPN is typically presenting in a stocking and glove pattern with the distal-most nerves affected first.

Evidence of the use of proteomics to study DPN is scarce. Studies on the proteome of dorsal root ganglion neurons from diabetic rats and appropriate controls suggest altered proteomes in the mitochondrion and dysfunction of the respiratory chain (Akude et al., 2011; Dobrowsky, 2016; Leal-Julià et al., 2022). Several challenges arise from studying the full proteome of a cell or tissue, such as high volumes of data, the complex nature of proteomes, and the dynamic nature of the proteome (Manzoni et al., 2018). We have previously described the full proteome of PIN in subjects with T2D, demonstrating early signs of DPN, and in healthy controls, using quantitative mass spectrometry (Ising et al., 2021).

The aim of this study is to present HSPs and describe their quantitative presentation in PINs from subjects with T1D, T2D, and healthy controls. Furthermore, this study also aimed to determine whether the protein quantities of specific HSPs are associated with T1D or T2D as well as to electrophysiology and morphometric data.

## 2. Methods

### 2.1. Subjects

A total of 56 subjects participated in this study and were allocated into three groups: T1D (*n* = 9; female = 6), T2D (*n* = 24; female = 7), and healthy controls without DM (*n* = 23; female = 12). Subjects were recruited from two previous cohorts. In total, 12 subjects (T2D: *n* = 9, controls: *n* = 3) were recruited from a large prospective health screening study in Malmö, Sweden (Eriksson et al., 1994). The rest of the subjects, in total *n* = 44, were recruited from a study on patients with carpal tunnel syndrome undergoing carpal tunnel release (Thomsen et al., 2009b). Out of the 44 subjects with carpal tunnel syndrome, 9 were diagnosed with T1D, 15 had T2D, and 20 were otherwise healthy. Characteristics, electrophysiology, and morphometry of data have been published previously but are gathered together in this study to describe the characteristics of the present cohort (Eriksson et al., 1994; Thomsen et al., 2009a,b; Osman et al., 2015; Ekman et al., 2020).

### 2.2. Biopsy of the posterior interosseous nerve

PINs were harvested under local or regional anesthesia, as described by Thomsen et al. (2009a). The harvested nerves were 3–4 cm in length and cut into four equally long pieces. Nerve specimens for mass spectrometry analysis were frozen fresh and kept at −80°C until solution digestion prior to liquid chromatography-tandem mass spectrometry (LC-MS/MS) analysis. The nerve specimen weighs ~1.15 mg per mm, and for LC-MS/MS analysis, each nerve specimen used was 5 mm long, i.e., 5.8 mg each.

### 2.3. Electrophysiology

Sural, peroneal, and ulnar nerves were used for electrophysiology assessment (Thomsen et al., 2009b). Parameters from the lower extremity include sural nerve sensory conduction velocity (sSCV), sural nerve amplitude (sAMP), and peroneal nerve motor conduction velocity (pMCV). Parameters from the upper extremity were obtained from the ulnar nerve and include sensory amplitude (uSAMP), sensory conduction velocity (uSCV), motor conduction velocity (uMCV), and motor amplitude at the wrist level (uAMP wrist).

### 2.4. Quantitative mass spectrometry: preparation and analysis

The full methodology of mass spectrometry sample preparation, LC-MS/MS analysis, and MS data analysis has been published previously (Ising et al., 2021). Fresh-frozen tissue pieces from PIN were used. In short, tissue pieces were buffered and homogenized and thereafter processed using trypsin prior to desalting and later LC-MS/MS analysis. Peptides were separated, and both data-dependent acquisition (DDA) and data-independent acquisition (DIA) were performed. Please refer to the article by Ising et al. for a detailed description of how DDA and DIA data were analyzed (Ising et al., 2021).

### 2.5. Statistical analyses

Characteristics are presented as median [quartiles] since data are not normally distributed. Differences in characteristics between groups are calculated using the overall Kruskal–Wallis test with the *post-hoc* Mann–Whitney U-test on significant values. The Mann–Whitney U-test was used when comparing only T1D and T2D. A *P*-value of < 0.05 is considered to be statistically significant. Statistical calculations of characteristics are made using IBM SPSS Statistics v. 27 for Mac (IBM Corp, Armonk, NY, USA).

The individual protein intensities obtained from biopsies of the 56 subjects were log2-transformed and normalized. Linear regression was used to discover which proteins were differentially expressed between T1D, T2D, and controls. We present both unadjusted regression results and adjusted regression results where we control for age, sex, and BMI. We also investigated the correlation between individual protein intensities and data from nerve conduction studies using Spearman's correlation. All *p*-values were adjusted for multiple comparisons using Benjamini and Hochberg (false discovery rate) correction. All statistical protein analyses were performed in R version 4.1 (R core team, R Foundation for Statistical Computing, Vienna, Austria).

## 3. Results

### 3.1. Subjects

The characteristics of all subjects (*n* = 56) divided into groups [T1D: *n* = 9 (female = 6); T2D: *n* = 24 (female = 7); and controls: *n* = 23 (female = 12)] are presented in Table 1. A total of 14 subjects with DM were treated with insulin, including all subjects with T1D and 5 out of 24 subjects with T2D. Subjects with T2D were generally older than T1D subjects and controls (Table 1), while the duration of DM among T2D subjects was significantly shorter (10 [3–15] years) compared to subjects with T1D (28 [13–38] years; *p* = 0.006).

**Table 1:** General characteristics. Characteristics are presented as median [quartiles] for each study group, i.e., controls, type 1 diabetes (T1D), and type 2 diabetes (T2D).

	**Controls (*n =* 23)**	**T1D (*n =* 9)**	**T2D (*n =* 24)**	**KW test^a^ (*p*-value)**	**Controls vs. T1D^b^ (*p-*value)**	**Controls vs. T2D^b^ (*p*-value)**	**T1D vs. T2D^b^ (*p*-value)**
Age (years)	57 [46–68]	46 [39–54]	68 [60–74]	< 0.001	0.025	0.009	< 0.001
Height (cm)	173 [166–180]	166 [162–176]	176 [165–178]	0.334	N/A	N/A	N/A
Weight (kg)	79 [70–88]	74 [57–78]	86 [82–94]	0.004	0.106	0.042	0.001
BMI (kg/m^2^)	26 [24–29]	25 [22–26]	29 [26–32]	0.004	0.068	0.020	0.005
Disease duration (years)		28 [13–38]	10 [3–15]^c^	N/A	N/A	N/A	0.003
HbA1c (mmol/mol)	26 [25–29]	58[55–78]	46 [36–57]	< 0.001	< 0.001	< 0.001	0.006
HbA1c (%)	4.5 [4.4–4.8]	7.5 [7.2–9.2]	6.4 [5.4–7.4]				

Characteristics between groups were compared using the Kruskal–Wallis test with the *post-hoc* Mann–Whitney U-test, and a *p*-value of < 0.05 was considered as statistically significant. Data have been published by Eriksson et al. (1994); Thomsen et al. (2009a,b), and Ekman et al. (2020).

N/A, not applicable.

^a^KW, Kruskal–Wallis test.

^b^Mann–Whitney U-test.

^c^*n* = 15.

HbA1c was found to be 26 [25–29] mmol/mol (4.5 [4.4–4.8] %) among controls, 58 [55–78] mmol/mol (7.5 [7.2–9.2] %) among subjects with T1D, and 46 [36–57] mmol/mol (6.4 [5.4–7.4] %) among subjects with T2D and differed between the groups (Kruskal–Wallis, *p* < 0.001). HbA1c was significantly higher in subjects with T1D compared to T2D and controls, as well as higher among subjects with T2D compared to controls.

### 3.2. PIN morphometry and protein expression

Myelinated nerve fiber density (MNFD) did not differ overall between groups [Kruskal–Wallis, p = 0.093; please observe that the present subjects were not identical to the ones presented by Thomsen et al. (2009b)].

The number of proteins was quantified using LC-MS/MS and the median [quartiles] number of proteins among controls, T1D subjects, and T2D subjects were 2540 [2516–2564], 2536 [2484–2562], and 2532 [2467–2553], respectively, with no difference between groups (Kruskal–Wallis, p = 0.645) (Table 2).

**Table 2:** Electrophysiology and PIN data.

	**Controls (*n =* 23)**	**T1D (*n =* 9)**	**T2D (*n =* 24)**	**KW test^a^ (*p*-value)**	**Controls vs. T1D^b^ (*p*-value)**	**Controls vs. T2D^b^ (*p*-value)**	**T1D vs. T2D^b^ (*p*-value)**
**Electrophysiology**							
sSCV (m/s)	45 [42.8–50]^c^	40 [34.5–47.5]	43 [40–44]^e^	0.007	0.029	0.004	0.435
sSAMP (μV)	8 [5–13]^c^	6 [2–14]^d^	3 [2–6]	0.001	0.358	< 0.001	0.209
pMCV (m/s)	46 [43–49]^c^	39 [34–43]	40 [37–45]	< 0.001	0.001	< 0.001	0.373
uSCV (m/s)	54 [48–56]^c^	52 [43–54]	48 [45–53]^e^	0.039	0.156	0.013	0.570
uSAMP (μV)	6 [3–8]^c^	3 [2–8]	4 [3–4]^*e*^	0.035	0.135	0.011	0.814
uMCV (m/s)	58 [54–62]^c^	52 [49–56]	56 [50–57]^e^	0.011	0.010	0.023	0.247
uAMP wrist (μV)	7 [6–9]^c^	8 [5.6–8.7]	6 [5–7]^e^	0.043	0.948	0.015	0.136
**Morphometrics**							
PIN MNFD (No./mm^2^)	5,893 [4,959–7,617]	4,575 [3,942–6,057]	5,356 [4,751–6,538]	0.093	N/A	N/A	N/A
**Proteomics**							
No. of proteins	2,540 [2,516–2,564]	2,536 [2,484–2,562]	2,532 [2,467–2,553]	0.645	N/A	N/A	N/A

Lower extremity parameters are sural nerve sensory conduction velocity (sSCV), sural nerve amplitude (sAMP), and peroneal nerve motor conduction velocity (pMCV). Parameters from the upper extremity are obtained from the ulnar nerve and include sensory amplitude (uSAMP), sensory conduction velocity (uSCV), motor conduction velocity (uMCV), and motor amplitude at the wrist level (uAMP wrist). Electrophysiology data are presented as median [quartiles] for each study group. The Kruskal–Wallis test with *post hoc* Mann–Whitney U-test was used to compare the groups. A *p*-value of < 0.05 was considered to be statistically significant. Electrophysiology and morphometrics data have been previously published by Eriksson et al. (1994); Thomsen et al. (2009a,b), and Ekman et al. (2020).

N/A, not applicable.

^a^KW, Kruskal–Wallis test.

^b^Mann–Whitney U-test.

^c^*n* = 22.

^d^*n* = 8.

^e^*n* = 23.

### 3.3. Electrophysiology

Electrophysiology data, for each group, are presented in Table 2 and showed differences between the groups (Kruskal–Wallis, *p* < 0.001–*p* = 0.043). Subjects with T1D had significantly lower conduction velocities in sural, peroneal, and ulnar nerves compared to controls. Subjects with T2D had significantly lower conduction velocities and amplitudes in all parameters compared to controls (Table 2).

### 3.4. Heat shock proteins

Heat shock proteins from all five heat shock families, including the human chaperonin family, were quantified (Kampinga et al., 2009; Stetler et al., 2010; de Los Reyes and Casas-Tintó, 2022). These families are HSP 70 superfamily (consisting of HSPA/HSP70 and HSPH/HSP110), the DNAJ family/HSP40 family, the HSPB/small heat shock proteins family, the HSPC/HSP90 family, and the chaperonin family (including HSPD/HSP60 and HSPE/HSP10). All identified HSPs, including putative HSPs, are presented in Table 3.

**Table 3:** Identified heat shock proteins by group.

**HSP 70 superfamily: HSPA (HSP 70) and HSPH (HSP110) families**			
**Subgroup**	**Name**	**Gene**	**Uniprot**
HSPA	Heat shock 70 kDa protein 1A (HSP70-1/HSP72)	HSPA1A	P0DMV8
	Heat shock 70 kDa protein 1-like	HSPA1L	P34931
	Heat shock-related 70 kDa protein 2	HSPA2	P54652
	Heat shock 70 kDa protein 4	HSPA4	P34932
	Heat shock 70 kDa protein 4L	HSPA4L	O95757
	Endoplasmic reticulum chaperone BiP	HSPA5	P11021
	Heat shock 70 kDa prote in 6	HSPA6	P17066
	Putative heat shock 70 kDa protein 7	HSPA7	P48741
	Heat shock cognate 71 kDa protein	HSPA8	P11142
	Stress-70 protein, mitochondrial	HSPA9	P38646
	Heat shock 70 kDa protein 12A	HSPA12A	O43301
	Heat shock 70 kDa protein 12B	HSPA12B	Q96MM6
HSPH	Heat shock protein 105 kDa	HSPH1	Q92598
**The DNAJ family (HSP40)**			
DnaJA	DnaJ homolog subfamily A member 2	DNAJA2	O60884
DnaJB	DnaJ homolog subfamily B member 2	DNAJB2	P25686
	DnaJ homolog subfamily B member 4	DNAJB4	Q9UDY4
DnaJC	DnaJ homolog subfamily C member 11	DNAJC11	Q9NVH1
**The HSPB family (small heat shock proteins)**			
HSPB	Heat shock protein beta-1 (HSP27)	HSPB1	P04792
	Heat shock protein beta-6	HSPB6	O14558
	Heat shock protein beta-8	HSPB8	Q9UJY1
**The HSP90/HSPC family**			
HSPC	Heat shock protein HSP 90-alpha	HSPC1/HSP90AA1	P07900
	Heat shock protein HSP 90-alpha A2	HSP90AA2P	Q14568
	Putative heat shock protein HSP 90-alpha A5	HSP90AA5P	Q58FG0
	Heat shock protein HSP 90-beta	HSPC3/HSP90AB1	P08238
	Putative heat shock protein HSP 90-beta 2	HSP90AB2P	Q58FF8
	Putative heat shock protein HSP 90-beta-3	HSP90AB3P	Q58FF7
	Putative heat shock protein HSP 90-beta 4	HSP90AB4P	Q58FF6
	Endoplasmin	HSPC4/HSP90B1	P14625
	Putative endoplasmin-like protein	HSP90B2P	Q58FF3
	Heat shock protein 75 kDa, mitochondrial (TRAP1)	HSPC5/TRAP1	Q12931
**Chaperonins and related genes**			
HSPD	60 kDa heat shock protein, mitochondrial	HSPD1	P10809
HSPE	10 kDa heat shock protein, mitochondrial	HSPE1	P61604

In total, 32 different heat shock proteins were identified among subjects with T1D, T2D, and controls. HSP90-alpha A5 was missing in one subject with T2D and HSP beta-8 was missing in six subjects with diabetes (T1D: *n* = 2; T2D: *n* = 4) but present in all other subjects.

All the present 32 HSPs were identified in all subjects except for putative heat shock protein HSP90-alpha A5 (Uniprot nr: Q58FG0) and HSP beta-8 (Uniprot nr: Q9UJY1). HSP90-alpha A5 was missing in one subject with T2D and HSP beta-8 was below the detection level in six subjects with diabetes (T1D: *n* = 2; T2D: *n* = 4) but present in substantially higher levels in all other subjects.

A linear regression model comparing each protein intensity between T1D, T2D, and controls could not demonstrate any statistical significance in the unadjusted model (Supplementary Table 1). Neither did a model adjusting for age, sex, and BMI (Supplementary Table 2) show any statistically significant difference when *p*-values had been adjusted for multiple comparisons (false discovery rate). However, several proteins of the HSP90 family (Uniprot nrs: Q58FF7, P08238, P07900, and Q58FF6), as well as HSP 70 (Uniprot nr: P0DMV8), approached statistical significance when studying the unadjusted *p*-values (Supplementary Table 1).

**Figure 1:**
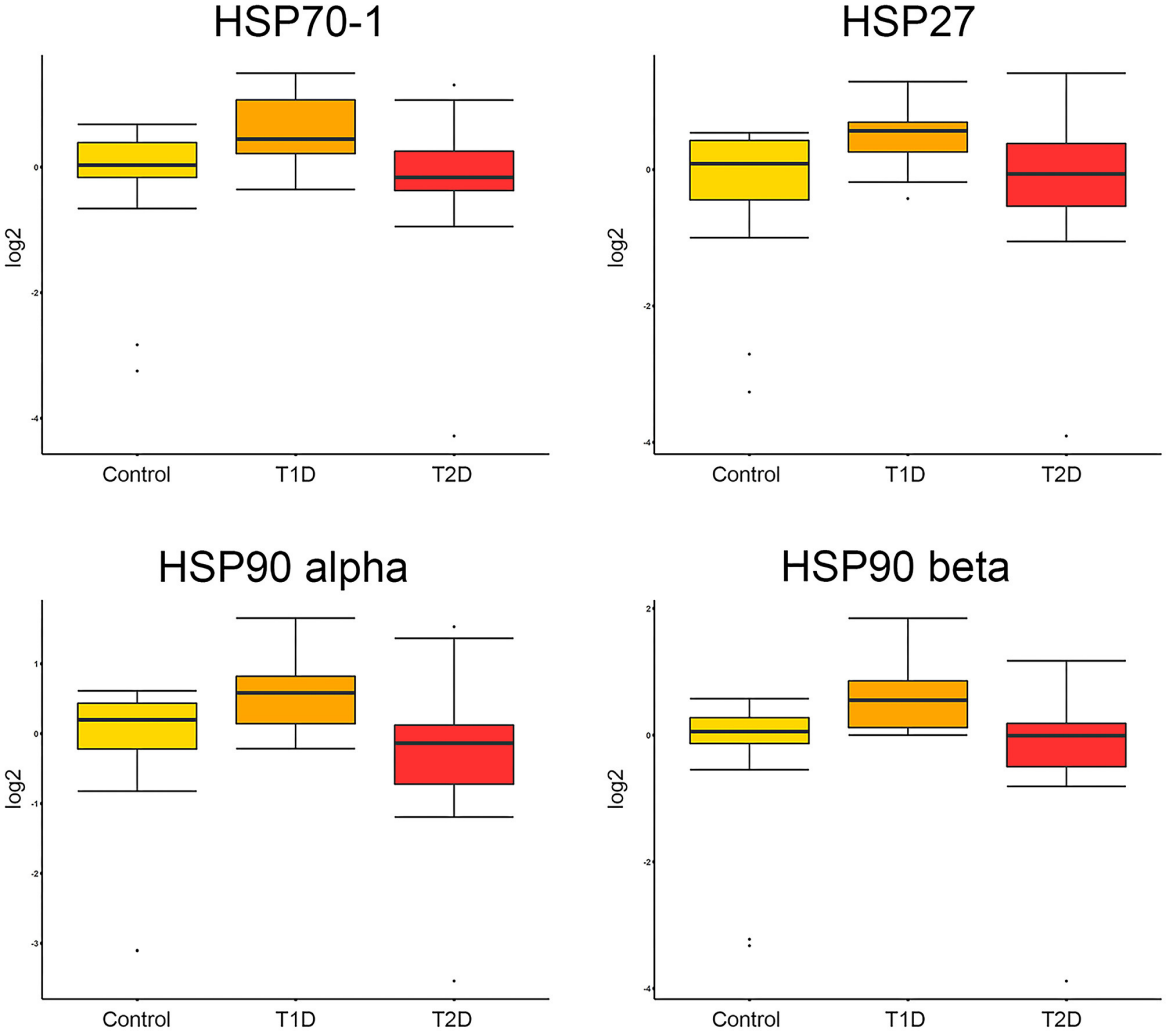
Boxplots of protein intensities. Protein intensities, log2-transformed and normalized of HSP70-1/HSP72, HSP27, HSP90-alpha, and HSP90 beta are presented as boxplots for each group of subjects. No statistically significant differences were found for any of the proteins above in the linear regression model when *p*-values were adjusted for multiple comparisons (Supplementary Table 1).

Boxplots of the distribution of protein intensities between groups are presented from three relevant proteins, with documented association to DPN, of different HSP families; i.e., HSP70 (Uniprot nr: P0DMV8), HSP90-alpha and HSP90 beta (Uniprot nrs: P07900 and P08238, respectively), and HSP beta-1/HSP27 (Uniprot nr: P04792) (Figure 1).

### 3.5. Correlations between protein intensities, electrophysiology, and morphometry

Using Spearman's correlations, individual protein intensities did not correlate with data from ulnar nerve conduction studies (i.e., uSAMP, uSCV, uMCV, and uAMP wrist) or morphometrics (MNFD) (data not presented graphically).

## 4. Discussion

The analysis of proteomics, in blood plasma/serum and nerve biopsies, is relevant when exploring the pathophysiology and etiology of diabetic neuropathy (Manzoni et al., 2018). HSPs have been implicated as important proteins in this context (Dobrowsky, 2016; Chaudhury et al., 2021). We have previously shown that HSPs can be identified using quantitative proteomics in biopsies of PIN from older male subjects with T2D (Ising et al., 2021). In this study, we demonstrate that the same HSPs can be identified in subjects with T1D. Furthermore, we can compare the intensities of 32 HSPs from the five HSP families between subjects with T1D and T2D and healthy controls.

In the present cohort, we found no statistically significant differences in the expression of the 32 HSPs in PIN from T1D subjects and T2D subjects compared to healthy controls when the *p*-values were adjusted for multiple comparisons. All controls were well established to be healthy through the normal oral glucose tolerance test. Although no clear tendencies were observed for any HSPs, comparing subjects with T1D to subjects with T2D or healthy controls, we speculate that intensities of HSPs could potentially be higher among subjects with T1D since the process of de- and re-generation is more pronounced in subjects with T1D (Osman et al., 2015). Such signs of regeneration are seen as regenerative clusters among such subjects at specific topographical locations in the nerve depending on the degeneration process (Dahlin et al., 2020). De- and re-generation after nerve injury and repair in healthy rats and in rats with diabetes (i.e., Goto-Kakizaki rats, resembling T2D) induce the upregulation of HSP27 both locally in the injured nerve as well as in the sensory neurons in the dorsal root ganglion (Stenberg et al., 2021). However, such increased local expression of HSP27 is not related to the outgrowth of nerve fibers, i.e., regeneration, after the nerve repair (Stenberg et al., 2021). Higher plasma levels of HSP27 have been found in T2D subjects with better nerve function and fewer signs of neuropathy (Pourhamidi et al., 2011) and are lower in T1D subjects with large nerve fiber dysfunction (Pourhamidi et al., 2014), indicating that HSP27, and potentially some other HSPs, is neuroprotective. However, contrary data have been published suggesting that HSP27 levels in plasma are higher among T1D subjects with DPN than in control subjects (Gruden et al., 2008). Interestingly, mild and severe hypoglycemia in T2D is also associated with changes in HSPs, such as HSP70 and HSP90, with an additional related inflammatory response to mild hypoglycemia among T2D subjects (Moin et al., 2021). These data indicate that the pattern of HSP expression in plasma and in the nervous system is complex in diabetes.

Although 32 HSPs were presented in our study, we neither observed any significant correlations to nerve conduction data from the ulnar nerve located at the same level as the PIN in the upper extremity nor we established any significant differences in protein intensities between subjects with T1D, T2D, and healthy controls. A possible explanation could be due to the heterogeneity of the subjects among groups of diabetes. For instance, there is a great range of disease duration among T1D and T2D subjects, where some subjects (T2D) had a short duration of diabetes. Thereby, the subjects with a short disease duration may impact the results on the group level due to the limited number of cases. Furthermore, old age and longer disease duration are often considered relevant for the development of DPN in T1D and T2D subjects (Hicks and Selvin, 2019). However, the median age among the present subjects was moderate in all groups in addition to a median disease duration among T2D subjects being not more than 10 years. Moreover, the subjects with diabetes have nerve conduction examinations that in most cases are within the normal range, and especially, the T2D subjects are meticulously controlled with mostly low HbA1c values. With that in mind, an extensive upregulation of HSPs in subjects with diabetes in our study could not be expected. For comparison, in the study by Gruden et al. mentioned above, HSP27 was higher in serum from T1D subjects with DPN compared to T1D subjects with no signs of complications to T1D, but both diabetes duration and age were higher among their subjects than the subjects in our study (Gruden et al., 2008). One may also argue that the degeneration process, observed as structural changes (Osman et al., 2015), may affect only specific topographical areas (Dahlin et al., 2020), resulting in lower neuroprotective compensation.

A limitation of this study is that the three groups are quite heterogenous, with skewness in the number of participants per group and a low number of subjects with T1D. In addition, a larger study population would reduce the risk of type 2 error. Furthermore, a limitation is that not all subjects with diabetes show electrophysiological signs of nerve dysfunction. Although the cohort is well-defined, there is a lack of data regarding insulin treatment for some of the T2D subjects. Such data could be relevant since HSPs have been shown to react to hypoglycemic events (Moin et al., 2021), and insulin-dependent T2D subjects are more prone to hypoglycemic events than non-insulin-dependent subjects.

In conclusion, we have demonstrated the presence of 32 HSPs, from all five HSP families, in human nerve biopsies of the upper extremity of subjects with T1D, T2D, and healthy controls. However, we did not find any difference in protein intensities between groups, as well as no association with nerve morphometry or nerve conduction findings. Nevertheless, we believe that the expression of HSPs in nerve biopsies needs further investigation. For example, to study the expression in cohorts with more extensive DPN compared to healthy controls without nerve dysfunction.

## Data availability statement

The datasets presented in this article are not readily available because public access to data is restricted to Swedish Authorities (Public Access to Information and Secrecy Act), but data can be available for researchers after a special review that includes approval of the research project by both an Ethics Committee and the Authorities' Data Safety Committees. Further inquiries can be directed to the corresponding author.

## Ethics statement

The studies involving human participants were reviewed and approved by the local Ethics Committee at Lund University, Lund, Sweden (LU508-03 and LU504-03). The patients/participants provided their written informed consent to participate in this study.

## Author contributions

EI and LD designed the study. LD and NT collected the patients and performed the surgery. EÅ and JM performed mass spectrometry. EI, JM, AÅ, and LD analyzed and interpreted data. EI drafted the manuscript. All authors have read and approved the final manuscript.

## References

[B1] Akude E. Zherebitskaya E. Chowdhury S. K. Smith D. R. Dobrowsky R. T. Fernyhough P.. (2011). Diminished superoxide generation is associated with respiratory chain dysfunction and changes in the mitochondrial proteome of sensory neurons from diabetic rats. Diabetes 60, 288–297. 10.2337/db10-0818 20876714 PMC3012184

[B2] Callaghan B. C. Little A. A. Feldman E. L. Hughes R. A. (2012). Enhanced glucose control for preventing and treating diabetic neuropathy. Cochrane Database Syst Rev (6), Cd007543. 10.1002/14651858.CD007543.pub2 22696371 PMC4048127

[B3] Chaudhury S. Keegan B. M. Blagg B. S. J. (2021). The role and therapeutic potential of Hsp90, Hsp70, and smaller heat shock proteins in peripheral and central neuropathies. Med. Res. Rev. 41, 202–222. 10.1002/med.21729 32844464 PMC8485878

[B4] Dahlin L. B. Rix K. R. Dahl V. A. Dahl A. B. Jensen J. N. Cloetens P.. (2020). Three-dimensional architecture of human diabetic peripheral nerves revealed by X-ray phase contrast holographic nanotomography. Sci. Rep. 10, 7592. 10.1038/s41598-020-64430-5 32371896 PMC7200696

[B5] de Los Reyes Casas-Tintó T. (2022). Neural functions of small heat shock proteins. Neural Regen Res 17, 512–515. 10.4103/1673-5374.320975 34380880 PMC8504394

[B6] Dobrowsky R. T. (2016). Targeting the diabetic chaperome to improve peripheral neuropathy. Curr. Diab. Rep. 16, 71. 10.1007/s11892-016-0769-8 27318486 PMC4929790

[B7] Ekman L. Thrainsdottir S. Englund E. Thomsen N. Rosén I. Hazer Rosberg D. B.. (2020). Evaluation of small nerve fiber dysfunction in type 2 diabetes. Acta Neurol. Scand. 141, 38–46. 10.1111/ane.13171 31549387

[B8] Eriksson K. F. Nilsson H. Lindgarde F. Osterlin S. Dahlin L. B. Lilja B.. (1994). Diabetes mellitus but not impaired glucose tolerance is associated with dysfunction in peripheral nerves. Diabet. Med. 11, 279–285. 8033527 10.1111/j.1464-5491.1994.tb00272.x

[B9] Farmer K. L. Li C. Dobrowsky R. T. (2012). Diabetic peripheral neuropathy: should a chaperone accompany our therapeutic approach? Pharmacol. Rev. 64, 880–900. 10.1124/pr.111.005314 22885705 PMC3462992

[B10] Feldman E. L. Callaghan B. C. Pop-Busui R. Zochodne D. W. Wright D. E. Bennett D. L.. (2019). Diabetic neuropathy. Nat Rev Dis Primers 5, 41. 10.1038/s41572-019-0092-1 31197153

[B11] Feldman E. L. Nave K. A. Jensen T. S. Bennett D. L. (2017). New Horizons in Diabetic Neuropathy: Mechanisms, Bioenergetics, and Pain. Neuron 93, 1296–1313. 10.1016/j.neuron.02005 28334605 PMC5400015

[B12] Gruden G. Bruno G. Chaturvedi N. Burt D. Schalkwijk C. Pinach S.. (2008). Serum heat shock protein 27 and diabetes complications in the EURODIAB prospective complications study. Novel Circ. Mark. Diab. Neuropathy 57, 1966–1970. 10.2337/db08-0009 18390793 PMC2453614

[B13] Hicks C. W. Selvin E. (2019). Epidemiology of peripheral neuropathy and lower extremity disease in diabetes. Curr. Diab. Rep. 19, 86. 10.1007/s11892-019-1212-8 31456118 PMC6755905

[B14] IDF (2021). “IDF Diabetes Atlas 10th edition. Brussels, Belgium: 2021”. Available online at: https://www.diabetesatlas.org:International/Diabetes/Federation

[B15] Ising E. Åhrman E. Thomsen N. O. B. Eriksson K. F. Malmström J. Dahlin L. B.. (2021). Quantitative proteomic analysis of human peripheral nerves from subjects with type 2 diabetes. Diabet Med. e14658. 10.1111./dme.14658 34309080

[B16] Jaiswal M. Divers J. Dabelea D. Isom S. Bell R. A. Martin C. L.. (2017). Prevalence of and risk factors for diabetic peripheral neuropathy in youth with type 1 and type 2 diabetes: search for diabetes in youth study. Diabetes Care 40, 1226–1232. 10.2337/dc17-0179 28674076 PMC5566278

[B17] Kampinga H. H. Hageman J. Vos M. J. Kubota H. Tanguay R. M. Bruford E. A.. (2009). Guidelines for the nomenclature of the human heat shock proteins. Cell Stress Chaperones 14, 105–111. 10.1007/s12192-008-0068-7 18663603 PMC2673902

[B18] Kerr M. Barron E. Chadwick P. Evans T. Kong W. M. Rayman G.. (2019). The cost of diabetic foot ulcers and amputations to the National Health Service in England. Diabet. Med. 36, 995–1002. 10.1111/dme.13973 31004370

[B19] Leal-Julià M. Vilches J. J. Onieva A. Verdés S. Sánchez Á. Chillón M.. (2022). Proteomic quantitative study of dorsal root ganglia and sciatic nerve in type 2 diabetic mice. Mol. Metabol. 55, 101408. 10.1016/j.molmet.2021.101408 34856394 PMC8717603

[B20] Malik R. A. (2014). “Chapter 18 - Pathology of human diabetic neuropathy,” in Handbook of Clinical Neurology, eds. Zochodne D.W. Malik R.A.. Elsevier, 249–259. 10.1016/B978-0-444-53480-4.00016-3 25410227

[B21] Malik R. A. Tesfaye S. Newrick P. G. Walker D. Rajbhandari S. M. Siddique I.. (2005). Sural nerve pathology in diabetic patients with minimal but progressive neuropathy. Diabetologia 48, 578–585. 10.1007/s00125-004-1663-5 15729579

[B22] Manzoni C. Kia D. A. Vandrovcova J. Hardy J. Wood N. W. Lewis P. A.. (2018). Genome, transcriptome and proteome: the rise of omics data and their integration in biomedical sciences. Brief. Bioinform. 19, 286–302. 10.1093/bib/bbw114 27881428 PMC6018996

[B23] Moin A. S. M. Nandakumar M. Kahal H. Sathyapalan T. Atkin S. L. Butler A. E.. (2021). Heat shock-related protein responses and inflammatory protein changes are associated with mild prolonged hypoglycemia. Cells 10, 3109. 10.3390./cells10113109 34831332 PMC8618421

[B24] Osman A. A. Dahlin L. B. Thomsen N. O. Mohseni S. (2015). Autophagy in the posterior interosseous nerve of patients with type 1 and type 2 diabetes mellitus: an ultrastructural study. Diabetologia 58, 625–632. 10.1007/s00125-014-3477-4 25523623

[B25] Pourhamidi K. Dahlin L. B. Boman K. Rolandsson O. (2011). Heat shock protein 27 is associated with better nerve function and fewer signs of neuropathy. Diabetologia 54, 3143–3149. 10.1007/s00125-011-2303-5 21909836

[B26] Pourhamidi K. Skärstrand H. Dahlin L. B. Rolandsson O. (2014). HSP27 concentrations are lower in patients with type 1 diabetes and correlate with large nerve fiber dysfunction. Diabetes Care 37, e49–50. 10.2337/dc13-1780 24558083

[B27] Rydberg M. Zimmerman M. Gottsäter A. Nilsson P. M. Melander O. Dahlin L. B.. (2020). Diabetes mellitus as a risk factor for compression neuropathy: a longitudinal cohort study from southern Sweden. BMJ Open Diabetes Res. Care 8, 1298. 10.1136./bmjdrc-2020-001298 32299900 PMC7199181

[B28] Stenberg L. Hazer Rosberg D. B. Kohyama S. Suganuma S. Dahlin L. B. (2021). Injury-Induced HSP27 expression in peripheral nervous tissue is not associated with any alteration in axonal outgrowth after immediate or delayed nerve repair. Int. J. Mol. Sci. 22, 8624. 10.3390./ijms22168624 34445330 PMC8395341

[B29] Stetler R. A. Gan Y. Zhang W. Liou A. K. Gao Y. Cao G.. (2010). (2010). Heat shock proteins: cellular and molecular mechanisms in the central nervous system. Prog. Neurobiol. 92, 184–211. 10.1016/j.pneurobio.05002 20685377 PMC2939168

[B30] Tesfaye S. Boulton A. J. Dyck P. J. Freeman R. Horowitz M. Kempler P.. (2010). Diabetic neuropathies: update on definitions, diagnostic criteria, estimation of severity, and treatments. Diabet. Care 33, 2285–2293. 10.2337/dc10-1303 20876709 PMC2945176

[B31] Thomsen N. O. Mojaddidi M. Malik R. A. Dahlin L. B. (2009a). Biopsy of the posterior interosseous nerve: a low morbidity method for assessment of peripheral nerve disorders. Diabet. Med. 26, 100–104. 10.1111/j.1464-5491.2008.02629.x 19125770

[B32] Thomsen N. O. Mojaddidi M. Malik R. A. Dahlin L. B. (2009b). Reduced myelinated nerve fibre and endoneurial capillary densities in the forearm of diabetic and non-diabetic patients with carpal tunnel syndrome. Acta Neuropathol. 118, 785–791. 10.1007/s00401-009-0578-0 19641929

[B33] Upton A. R. McComas A. J. (1973). The double crush in nerve entrapment syndromes. Lancet 2, 359–362. 10.1016/s0140-6736(73)93196-6 4124532

[B34] Urban M. J. Pan P. Farmer K. L. Zhao H. Blagg B. S. Dobrowsky R. T.. (2012). Modulating molecular chaperones improves sensory fiber recovery and mitochondrial function in diabetic peripheral neuropathy. Exp. Neurol. 235, 388–396. 10.1016/j.expneurol.03005 22465570 PMC3336191

