# Supplementary material for: Quantification of heat shock proteins in the posterior interosseous nerve among subjects with type 1 and type 2 diabetes compared to healthy controls
Source: Front Neurosci. 2023 Aug 8;17:1227557. doi: 10.3389/fnins.2023.1227557 (PMC10442572; doi:10.3389/fnins.2023.1227557)
Supplement: Supplementary file 1 [file Data_Sheet_1.docx]

Supplementary Table 1. Results from linear regressions models comparing each protein evaluated in human posterior interosseous nerves in T1D, T2D, and controls.

| **Name** | **Family** | **Gene** | **Uniprot** |  | **β (95% CI)** | **Adj.p-val*** |
| --- | --- | --- | --- | --- | --- | --- |
| Putative heat shock protein HSP 90-beta-3 | HSP90/HSPC | HSP90AB3P | Q58FF7 | Group |  | 0.246 |
|  |  |  |  | Control | Ref. |  |
|  |  |  |  | T1D | 0.870 (0.206 ; 1.530) |  |
|  |  |  |  | T2D | 0.172 (-0.387 ; 0.731) |  |
| Heat shock protein  HSP 90-beta | HSP90/HSPC | HSP90AB1 | P08238 | Group |  | 0.246 |
|  |  |  |  | Control | Ref. |  |
|  |  |  |  | T1D | 0.801 (0.128 ; 1.470) |  |
|  |  |  |  | T2D | 0.043 (-0.525 ; 0.610) |  |
| Heat shock protein  HSP 90-alpha | HSP90/HSPC | HSP90AA1 | P07900 | Group |  | 0.246 |
|  |  |  |  | Control | Ref. |  |
|  |  |  |  | T1D | 0.737 (0.06 ; 1.410) |  |
|  |  |  |  | T2D | -0.077 (-0.647 ; 0.492) |  |
| Putative heat shock protein HSP 90-beta 4 | HSP90/HSPC | HSP90AB4P | Q58FF6 | Group |  | 0.246 |
|  |  |  |  | Control | Ref. |  |
|  |  |  |  | T1D | 0.776 (0.102 ; 1.450) |  |
|  |  |  |  | T2D | 0.013 (-0.556 ; 0.582) |  |
| Heat shock cognate 71 kDa protein | HSP70 superfamily (HSPA/HSPH) | HSPA8 | P11142 | Group |  | 0.246 |
|  |  |  |  | Control | Ref. |  |
|  |  |  |  | T1D | 0.819 (0.143 ; 1.500) |  |
|  |  |  |  | T2D | 0.141 (-0.430 ; 0.711) |  |
| Heat shock 70 kDa protein 6 | HSP70 superfamily (HSPA/HSPH) | HSPA6 | P17066 | Group |  | 0.246 |
|  |  |  |  | Control | Ref. |  |
|  |  |  |  | T1D | 0.726 (0.048 ; 1.400) |  |
|  |  |  |  | T2D | -0.062 (-0.634 ; 0.51) |  |
| Putative heat shock 70 kDa protein 7 | HSP70 superfamily (HSPA/HSPH) | HSPA7 | P48741 | Group |  | 0.246 |
|  |  |  |  | Control | Ref. |  |
|  |  |  |  | T1D | 0.739 (0.046 ; 1.430) |  |
|  |  |  |  | T2D | -0.028 (-0.612 ; 0.557) |  |
| Heat shock 70 kDa protein 1A | HSP70 superfamily (HSPA/HSPH) | HSPA1A | P0DMV8 | Group |  | 0.246 |
|  |  |  |  | Control | Ref. |  |
|  |  |  |  | T1D | 0.737 (0.053 ; 1.42) |  |
|  |  |  |  | T2D | -0.008 (-0.585 ; 0.569) |  |
| Putative heat shock protein HSP 90-beta 2 | HSP90/HSPC | HSP90AB2P | Q58FF8 | Group |  | 0.246 |
|  |  |  |  | Control | Ref. |  |
|  |  |  |  | T1D | 0.739 (0.059 ; 1.420) |  |
|  |  |  |  | T2D | 0.040 (-0.534 ; 0.613) |  |
| Heat shock protein beta-1 | Small HSPs/HSPB | HSPB1 | P04792 | Group |  | 0.246 |
|  |  |  |  | Control | Ref. |  |
|  |  |  |  | T1D | 0.741 (0.072 ; 1.410) |  |
|  |  |  |  | T2D | 0.103 (-0.460 ; 0.667) |  |
| Endoplasmin | HSP90/HSPC | HSP90B1 | P14625 | Group |  | 0.246 |
|  |  |  |  | Control | Ref. |  |
|  |  |  |  | T1D | 0.712 (0.026 ; 1.400) |  |
|  |  |  |  | T2D | 0.022 (-0.556 ; 0.600) |  |
| DnaJ homolog subfamily B member 4 | HSP40/DNAJ | DNAJB4 | Q9UDY4 | Group |  | 0.246 |
|  |  |  |  | Control | Ref. |  |
|  |  |  |  | T1D | 0.484 (-0.210 ; 1.180) |  |
|  |  |  |  | T2D | -0.295 (-0.881 ; 0.291) |  |
| Stress-70 protein, mitochondrial | HSP70 superfamily (HSPA/HSPH) | HSPA9 | P38646 | Group |  | 0.246 |
|  |  |  |  | Control | Ref. |  |
|  |  |  |  | T1D | 0.699 (0.005 ; 1.390) |  |
|  |  |  |  | T2D | -0.003 (-0.588 ; 0.582) |  |
| DnaJ homolog subfamily A member 2 | HSP40/DNAJ | DNAJA2 | O60884 | Group |  | 0.273 |
|  |  |  |  | Control | Ref. |  |
|  |  |  |  | T1D | 0.522 (-0.127 ; 1.170) |  |
|  |  |  |  | T2D | -0.152 (-0.699 ; 0.395) |  |
| Heat shock protein 75 kDa, mitochondrial | HSP90/HSPC | TRAP1 | Q12931 | Group |  | 0.273 |
|  |  |  |  | Control | Ref. |  |
|  |  |  |  | T1D | 0.518 (-0.173 ; 1.210) |  |
|  |  |  |  | T2D | -0.203 (-0.786 ; 0.380) |  |
| Heat shock-related 70 kDa protein 2 | HSP70 superfamily (HSPA/HSPH) | HSPA2 | P54652 | Group |  | 0.280 |
|  |  |  |  | Control | Ref. |  |
|  |  |  |  | T1D | 0.686 (0.004 ; 1.370) |  |
|  |  |  |  | T2D | 0.131 (-0.444 ; 0.706) |  |
| Heat shock protein  HSP 90-alpha A2 | HSP90/HSPC | HSP90AA2P | Q14568 | Group |  | 0.281 |
|  |  |  |  | Control | Ref. |  |
|  |  |  |  | T1D | 0.648 (-0.042 ; 1.340) |  |
|  |  |  |  | T2D | 0.040 (-0.542 ; 0.621) |  |
| Heat shock 70 kDa protein 12A | HSP70 superfamily (HSPA/HSPH) | HSPA12A | O43301 | Group |  | 0.281 |
|  |  |  |  | Control | Ref. |  |
|  |  |  |  | T1D | 0.563 (-0.131 ; 1.260) |  |
|  |  |  |  | T2D | -0.103 (-0.688 ; 0.483) |  |
| Putative heat shock protein HSP 90-alpha A5 | HSP90/HSPC | HSP90AA5P | Q58FG0 | Group |  | 0.333 |
|  |  |  |  | Control | Ref. |  |
|  |  |  |  | T1D | 0.334 (-0.370 ; 1.040) |  |
|  |  |  |  | T2D | -0.309 (-0.903 ; 0.285) |  |
| 60 kDa heat shock protein, mitochondrial | Chaperonins (HSPE/D) | HSPD1 | P10809 | Group |  | 0.354 |
|  |  |  |  | Control | Ref. |  |
|  |  |  |  | T1D | 0.545 (-0.161 ; 1.250) |  |
|  |  |  |  | T2D | -0.049 (-0.644 ; 0.547) |  |
| 10 kDa heat shock protein, mitochondrial | Chaperonins (HSPE/D) | HSPE1 | P61604 | Group |  | 0.381 |
|  |  |  |  | Control | Ref. |  |
|  |  |  |  | T1D | 0.552 (-0.150 ; 1.250) |  |
|  |  |  |  | T2D | 0.016 (-0.575 ; 0.608) |  |
| Endoplasmic reticulum chaperone BiP | HSP70 superfamily (HSPA/HSPH) | HSPA5 | P11021 | Group |  | 0.381 |
|  |  |  |  | Control | Ref. |  |
|  |  |  |  | T1D | 0.566 (-0.132 ; 1.260) |  |
|  |  |  |  | T2D | 0.114 (-0.475 ; 0.703) |  |
| Heat shock protein beta-8 | Small HSPs/HSPB | HSPB8 | Q9UJY1 | Group |  | 0.381 |
|  |  |  |  | Control | Ref. |  |
|  |  |  |  | T1D | -0.499 (-1.170 ; 0.172) |  |
|  |  |  |  | T2D | -0.366 (-0.932 ; 0.200) |  |
| Heat shock 70 kDa protein 1-like | HSP70 superfamily (HSPA/HSPH) | HSPA1L | P34931 | Group |  | 0.381 |
|  |  |  |  | Control | Ref. |  |
|  |  |  |  | T1D | 0.545 (-0.151 ; 1.240) |  |
|  |  |  |  | T2D | 0.076 (-0.510 ; 0.662) |  |
| Heat shock protein beta-6 | Small HSPs/HSPB | HSPB6 | O14558 | Group |  | 0.465 |
|  |  |  |  | Control | Ref. |  |
|  |  |  |  | T1D | 0.388 (-0.326 ; 1.100) |  |
|  |  |  |  | T2D | -0.118 (-0.720 ; 0.484) |  |
| Putative endoplasmin-like protein | HSP90/HSPC | HSP90B2P | Q58FF3 | Group |  | 0.465 |
|  |  |  |  | Control | Ref. |  |
|  |  |  |  | T1D | 0.495 (-0.221 ; 1.210) |  |
|  |  |  |  | T2D | 0.086 (-0.518 ; 0.69) |  |
| Heat shock 70 kDa protein 4 | HSP70 superfamily (HSPA/HSPH) | HSPA4 | P34932 | Group |  | 0.465 |
|  |  |  |  | Control | Ref. |  |
|  |  |  |  | T1D | 0.331 (-0.384 ; 1.050) |  |
|  |  |  |  | T2D | -0.169 (-0.772 ; 0.434) |  |
| DnaJ homolog subfamily B member 2 | HSP40/DNAJ | DNAJB2 | P25686 | Group |  | 0.477 |
|  |  |  |  | Control | Ref. |  |
|  |  |  |  | T1D | 0.440 (-0.220 ; 1.100) |  |
|  |  |  |  | T2D | 0.089 (-0.468 ; 0.645) |  |
| Heat shock 70 kDa protein 12B | HSP70 superfamily (HSPA/HSPH) | HSPA12B | Q96MM6 | Group |  | 0.536 |
|  |  |  |  | Control | Ref. |  |
|  |  |  |  | T1D | 0.441 (-0.277 ; 1.160) |  |
|  |  |  |  | T2D | 0.128 (-0.478 ; 0.734) |  |
| DnaJ homolog subfamily C member 11 | HSP40/DNAJ | DNAJC11 | Q9NVH1 | Group |  | 0.613 |
|  |  |  |  | Control | Ref. |  |
|  |  |  |  | T1D | -0.389 (-1.110 ; 0.333) |  |
|  |  |  |  | T2D | -0.135 (-0.743 ; 0.473) |  |
| Heat shock 70 kDa protein 4L | HSP70 superfamily (HSPA/HSPH) | HSPA4L | O95757 | Group |  | 0.613 |
|  |  |  |  | Control | Ref. |  |
|  |  |  |  | T1D | 0.278 (-0.435 ; 0.991) |  |
|  |  |  |  | T2D | -0.089 (-0.690 ; 0.513) |  |
| Heat shock protein 105 kDa | HSP70 superfamily (HSPA/HSPH) | HSPH1 | Q92598 | Group |  | 0.631 |
|  |  |  |  | Control | Ref. |  |
|  |  |  |  | T1D | 0.136 (-0.581 ; 0.853) |  |
|  |  |  |  | T2D | -0.198 (-0.802 ; 0.407) |  |

*****Adjusted for multiple coparisons.

Supplementary Table 2. Results from linear regressions models comparing each protein evaluated in human posterior interosseous nerves in T1D, T2D, and controls, adjusting for age, sex, and BMI.

| **Name** | **Family** | **Gene** | **Uniprot** |  | **β (95% CI)** | **Adj.p-val*** |
| --- | --- | --- | --- | --- | --- | --- |
| Putative heat shock protein  HSP 90-beta-3 | HSP90/HSPC | HSP90AB3P | Q58FF7 | Group |  | 0.585 |
|  |  |  |  | Control | Ref. |  |
|  |  |  |  | T1D | 0.762 (0.040 ; 1.480) |  |
|  |  |  |  | T2D | 0.267 (-0.371 ; 0.905) |  |
| Heat shock cognate 71 kDa protein | HSP70 superfamily (HSPA/HSPH) | HSPA8 | P11142 | Group |  | 0.585 |
|  |  |  |  | Control | Ref. |  |
|  |  |  |  | T1D | 0.765 (0.024 ; 1.510) |  |
|  |  |  |  | T2D | 0.185 (-0.470 ; 0.840) |  |
| Putative heat shock protein  HSP 90-beta 4 | HSP90/HSPC | HSP90AB4P | Q58FF6 | Group |  | 0.585 |
|  |  |  |  | Control | Ref. |  |
|  |  |  |  | T1D | 0.700 (-0.030 ; 1.430) |  |
|  |  |  |  | T2D | 0.049 (-0.596 ; 0.695) |  |
| Heat shock protein HSP 90-beta | HSP90/HSPC | HSP90AB1 | P08238 | Group |  | 0.585 |
|  |  |  |  | Control | Ref. |  |
|  |  |  |  | T1D | 0.688 (-0.040 ; 1.420) |  |
|  |  |  |  | T2D | 0.131 (-0.512 ; 0.774) |  |
| Putative heat shock protein  HSP 90-beta 2 | HSP90/HSPC | HSP90AB2P | Q58FF8 | Group |  | 0.585 |
|  |  |  |  | Control | Ref. |  |
|  |  |  |  | T1D | 0.662 (-0.075 ; 1.400) |  |
|  |  |  |  | T2D | 0.081 (-0.571 ; 0.732) |  |
| Heat shock protein beta-1 | Small HSPs/HSPB | HSPB1 | P04792 | Group |  | 0.585 |
|  |  |  |  | Control | Ref. |  |
|  |  |  |  | T1D | 0.641 (-0.077 ; 1.360) |  |
|  |  |  |  | T2D | 0.149 (-0.485 ; 0.783) |  |
| Heat shock protein  HSP 90-alpha | HSP90/HSPC | HSP90AA1 | P07900 | Group |  | 0.585 |
|  |  |  |  | Control | Ref. |  |
|  |  |  |  | T1D | 0.626 (-0.098 ; 1.350) |  |
|  |  |  |  | T2D | 0.002 (-0.638 ; 0.641) |  |
| Endoplasmin | HSP90/HSPC | HSP90B1 | P14625 | Group |  | 0.585 |
|  |  |  |  | Control | Ref. |  |
|  |  |  |  | T1D | 0.653 (-0.099 ; 1.400) |  |
|  |  |  |  | T2D | 0.077 (-0.587 ; 0.742) |  |
| Heat shock-related 70 kDa protein 2 | HSP70 superfamily (HSPA/HSPH) | HSPA2 | P54652 | Group |  | 0.585 |
|  |  |  |  | Control |  |  |
|  |  |  |  | T1D | 0.643 (-0.102 ; 1.390) |  |
|  |  |  |  | T2D | 0.147 (-0.513 ; 0.806) |  |
| Putative heat shock 70 kDa protein 7 | HSP70 superfamily (HSPA/HSPH) | HSPA7 | P48741 | Group |  | 0.585 |
|  |  |  |  | Control | Ref. |  |
|  |  |  |  | T1D | 0.646 (-0.112 ; 1.400) |  |
|  |  |  |  | T2D | 0.058 (-0.611 ; 0.728) |  |
| DnaJ homolog subfamily B member 4 | HSP40/DNAJ | DNAJB4 | Q9UDY4 | Group |  | 0.585 |
|  |  |  |  | Control | Ref. |  |
|  |  |  |  | T1D | 0.475 (-0.270 ; 1.220) |  |
|  |  |  |  | T2D | -0.261 (-0.92 ; 0.398) |  |
| Heat shock protein beta-8 | Small HSPs/HSPB | HSPB8 | Q9UJY1 | Group |  | 0.585 |
|  |  |  |  | Control | Ref. |  |
|  |  |  |  | T1D | -0.570 (-1.280 ; 0.1430) |  |
|  |  |  |  | T2D | -0.265 (-0.895 ; 0.365) |  |
| Heat shock 70 kDa protein 6 | HSP70 superfamily (HSPA/HSPH) | HSPA6 | P17066 | Group |  | 0.585 |
|  |  |  |  | Control | Ref. |  |
|  |  |  |  | T1D | 0.614 (-0.127 ; 1.350) |  |
|  |  |  |  | T2D | 0.062 (-0.592 ; 0.716) |  |
| Heat shock 70 kDa protein 1A | HSP70 superfamily (HSPA/HSPH) | HSPA1A | P0DMV8 | Group |  | 0.585 |
|  |  |  |  | Control | Ref. |  |
|  |  |  |  | T1D | 0.600 (-0.140 ; 1.340) |  |
|  |  |  |  | T2D | 0.109 (-0.546 ; 0.763) |  |
| Putative heat shock protein HSP 90-alpha A5 | HSP90/HSPC | HSP90AA5P | Q58FG0 | Group |  | 0.585 |
|  |  |  |  | Control | Ref. |  |
|  |  |  |  | T1D | 0.374 (-0.392 ; 1.140) |  |
|  |  |  |  | T2D | -0.354 (-1.030 ; 0.324) |  |
| Heat shock protein  HSP 90-alpha A2 | HSP90/HSPC | HSP90AA2P | Q14568 | Group |  | 0.585 |
|  |  |  |  | Control | Ref. |  |
|  |  |  |  | T1D | 0.588 (-0.160 ; 1.340) |  |
|  |  |  |  | T2D | 0.062 (-0.598 ; 0.723) |  |
| Stress-70 protein, mitochondrial | HSP70 superfamily (HSPA/HSPH) | HSPA9 | P38646 | Group |  | 0.585 |
|  |  |  |  | Control | Ref. |  |
|  |  |  |  | T1D | 0.574 (-0.163 ; 1.310) |  |
|  |  |  |  | T2D | 0.054 (-0.597 ; 0.706) |  |
| 10 kDa heat shock protein, mitochondrial | Chaperonins (HSPE/D) | HSPE1 | P61604 | Group |  | 0.585 |
|  |  |  |  | Control | Ref. |  |
|  |  |  |  | T1D | 0.527 (-0.225 ; 1.280) |  |
|  |  |  |  | T2D | 0.118 (-0.547 ; 0.783) |  |
| Putative endoplasmin-like protein | HSP90/HSPC | HSP90B2P | Q58FF3 | Group |  | 0.585 |
|  |  |  |  | Control | Ref. |  |
|  |  |  |  | T1D | 0.514 (-0.250 ; 1.280) |  |
|  |  |  |  | T2D | -0.018 (-0.693 ; 0.657) |  |
| Endoplasmic reticulum chaperone BiP | HSP70 superfamily (HSPA/HSPH) | HSPA5 | P11021 | Group |  | 0.585 |
|  |  |  |  | Control | Ref. |  |
|  |  |  |  | T1D | 0.516 (-0.239 ; 1.270) |  |
|  |  |  |  | T2D | 0.109 (-0.558 ; 0.776) |  |
| Heat shock 70 kDa protein 12A | HSP70 superfamily (HSPA/HSPH) | HSPA12A | O43301 | Group |  | 0.585 |
|  |  |  |  | Control | Ref. |  |
|  |  |  |  | T1D | 0.483 (-0.272 ; 1.240) |  |
|  |  |  |  | T2D | -0.056 (-0.723 ; 0.612) |  |
| 60 kDa heat shock protein, mitochondrial | Chaperonins (HSPE/D) | HSPD1 | P10809 | Group |  | 0.585 |
|  |  |  |  | Control | Ref. |  |
|  |  |  |  | T1D | 0.483 (-0.273 ; 1.240) |  |
|  |  |  |  | T2D | -0.051 (-0.719 ; 0.618) |  |
| Heat shock 70 kDa protein 12B | HSP70 superfamily (HSPA/HSPH) | HSPA12B | Q96MM6 | Group |  | 0.585 |
|  |  |  |  | Control | Ref. |  |
|  |  |  |  | T1D | 0.514 (-0.266 ; 1.290) |  |
|  |  |  |  | T2D | 0.096 (-0.593 ; 0.785) |  |
| Heat shock protein beta-6 | Small HSPs/HSPB | HSPB6 | O14558 | Group |  | 0.585 |
|  |  |  |  | Control | Ref. |  |
|  |  |  |  | T1D | 0.432 (-0.350 ; 1.210) |  |
|  |  |  |  | T2D | -0.157 (-0.848 ; 0.535) |  |
| DnaJ homolog subfamily B member 2 | HSP40/DNAJ | DNAJB2 | P25686 | Group |  | 0.585 |
|  |  |  |  | Control | Ref. |  |
|  |  |  |  | T1D | 0.448 (-0.260 ; 1.160) |  |
|  |  |  |  | T2D | 0.016 (-0.613 ; 0.638) |  |
| Heat shock 70 kDa protein 1-like | HSP70 superfamily (HSPA/HSPH) | HSPA1L | P34931 | Group |  | 0.628 |
|  |  |  |  | Control | Ref. |  |
|  |  |  |  | T1D | 0.444 (-0.306 ; 1.190) |  |
|  |  |  |  | T2D | 0.132 (-0.531 ; 0.795) |  |
| DnaJ homolog subfamily C member 11 | HSP40/DNAJ | DNAJC11 | Q9NVH1 | Group |  | 0.649 |
|  |  |  |  | Control | Ref. |  |
|  |  |  |  | T1D | -0.440 (-1.220 ; 0.344) |  |
|  |  |  |  | T2D | -0.053 (-0.746 ; 0.640) |  |
| Heat shock protein 75 kDa, mitochondrial | HSP90/HSPC | TRAP1 | Q12931 | Group |  | 0.702 |
|  |  |  |  | Control | Ref. |  |
|  |  |  |  | T1D | 0.345 (-0.353 ; 1.040) |  |
|  |  |  |  | T2D | 0.043 (-0.574 ; 0.659) |  |
| DnaJ homolog subfamily A member 2 | HSP40/DNAJ | DNAJA2 | O60884 | Group |  | 0.702 |
|  |  |  |  | Control | Ref. |  |
|  |  |  |  | T1D | 0.313 (-0.355 ; 0.980) |  |
|  |  |  |  | T2D | 0.137 (-0.452 ; 0.727) |  |
| Heat shock 70 kDa protein 4L | HSP70 superfamily (HSPA/HSPH) | HSPA4L | O95757 | Group |  | 0.899 |
|  |  |  |  | Control | Ref. |  |
|  |  |  |  | T1D | 0.050 (-0.705 ; 0.805) |  |
|  |  |  |  | T2D | 0.199 (-0.468 ; 0.866) |  |
| Heat shock 70 kDa protein 4 | HSP70 superfamily (HSPA/HSPH) | HSPA4 | P34932 | Group |  | 0.949 |
|  |  |  |  | Control | Ref. |  |
|  |  |  |  | T1D | 0.108 (-0.650 ; 0.865) |  |
|  |  |  |  | T2D | 0.121 (-0.549 ; 0.791) |  |
| Heat shock protein 105 kDa | HSP70 superfamily (HSPA/HSPH) | HSPH1 | Q92598 | Group |  | 0.998 |
|  |  |  |  | Control | Ref. |  |
|  |  |  |  | T1D | 0.021 (-0.720 ; 0.761) |  |
|  |  |  |  | T2D | -0.006 (-0.661 ; 0.648) |  |
